# Anatomic Combined Anterior Cruciate Ligament and Antero-Lateral Ligament Reconstruction Using Autologous Gracilis and Semitendinosus Graft With Single Tibial and Femoral Tunnel

**DOI:** 10.1016/j.eats.2022.10.015

**Published:** 2023-01-18

**Authors:** Daniele Screpis, Marco Baldini, Stefano Magnanelli, Andrea Amarossi, Gianluca Piovan, Simone Natali, Claudio Zorzi

**Affiliations:** a IRCCS Ospedale Sacro Cuore - Don Calabria, Negrar di Valpolicella, Italia; b Clinica Ortopedica dell’Adulto e Pediatrica, Università Politecnica delle Marche, Ancona, Italia; c Department of Orthopedics and Trauma Surgery, University of Verona, Verona, Italy

## Abstract

Anterior cruciate ligament (ACL) injuries are among the most common lesions in orthopaedics practice, but still today, rates up to 24% of unsatisfactory results are reported. Unaddressed anterolateral complex (ALC) injuries have been claimed to be responsible for residual anterolateral rotatory instability (ALRI) after isolate ACL reconstruction and have demonstrated to increase graft failure. In this article, we present our technique for reconstruction of the ACL and anterolateral (ALL) ligament combining the advantages of the anatomical position and the intraosseous femoral fixation to ensure anteroposterior and anterolateral rotational stability.

## Introduction

Anterior cruciate ligament (ACL) injuries are among the most common lesions in orthopaedic practice, with an estimated incidence in the United States (US) ranging from 30 to 78 per 100,000 person/years.

Even if ACL reconstruction is considered by many inexperienced surgeons to be a “simple procedure”, still today, only 82% of patients are able to return to sport. When considering return to preinjury level (63%) or competitive sports (44%) percentage are even lower,^1^ with some reporting up to 24% of unsatisfactory results, and 10% of failure rates at follow-up.^2^^,^^3^

Even if there is still some debate on the existence and function of the anterolateral ligament (ALL), numerous authors demonstrated the ability to consistently identify this structure in nearly all analyzed specimens using specific anatomical dissection technique.^4^^,^^5^^,^^6^^,^^7^

Moreover, biomechanical studies confirmed that the anterolateral complex (ALC) acts as an important anatomical constraint against tibial internal rotation.^8^ Thus, undiagnosed and untreated ALC injury have been claimed to be responsible for residual anteroposterior and rotational instability that may affect up to 25% of patients after isolated ACL reconstruction.^9^^,^^10^ Indeed, recent biomechanical studies confirmed the reduction of in situ forces on ACL graft after LET (lateral extra-articular tenodesis). Risk factors for residual anterolateral rotatory instability (ALRI) after ACLR include joint hyperlaxity and a high-grade clinical pivot-shift.^11^ In addition, high-demand athletes involved in pivoting sports and ACL revision surgery are increasingly accepted as indication for additional extra-articular surgical procedure targeting ALC complex, given that ALL reconstruction is associated with significantly reduced graft ruptures.^12^^,^^13^

Although different techniques have been proposed for reconstruction of ALL, consensus on which is to be considered the best is lacking, even if it is well accepted that anatomical reconstruction is to be addressed whenever possible.^14^ In this article, we present our technique for anatomical combined ACL and ALL reconstruction using single-stranded gracilis (GR) and semitendinosus (ST) autograft with intact distal insertion, requiring only one tibial and one femoral tunnel.

## Surgical Technique

### Patient Positioning

The patient is placed supine in a standard arthroscopic position with a lateral leg holder and a vertical post at the level of the thigh, just proximal to the knee. Leg holder is positioned in a way that allows nearly full flexion of the knee during the entire procedure. An inflatable tourniquet is placed at the base of the thigh.

### Graft Harvesting and Preparation

GR and ST are harvested using an open-ended stripper and measured, after removing muscular tissue. Total length of the graft of at least 16 cm is required to perform this procedure.

**Fig 1:**
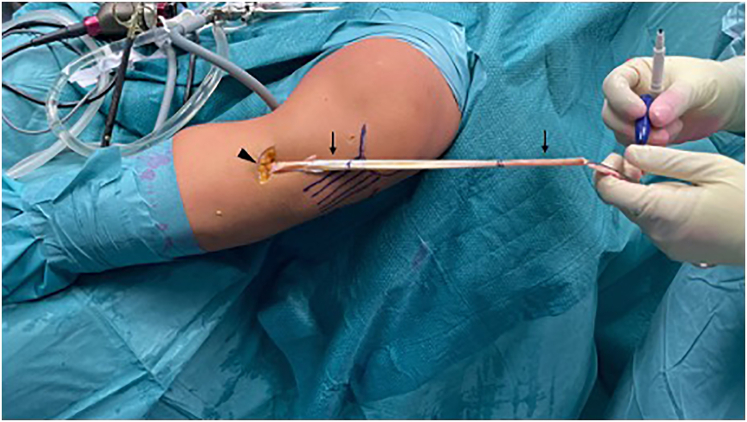
Graft preparation. The graft is made of single stranded GR and ST with intact distal insertions (arrowhead). Both extremities are then whipstitched with high-resistance sutures (arrows). GR, gracilis; ST, semitendinosus.

The tendons are whipstitched together near the distal insertion (Vycril 2, Ethicon) and at the free proximal end with a high-resistance nonabsorbable suture (FiberWire 2, Arthrex) (Fig 1).

### Tunnel Positioning and Landmarks

After arthroscopic evaluation and eventual treatment of associated intraarticular lesions, femoral and tibial tunnel are drilled.

**Fig 2:**
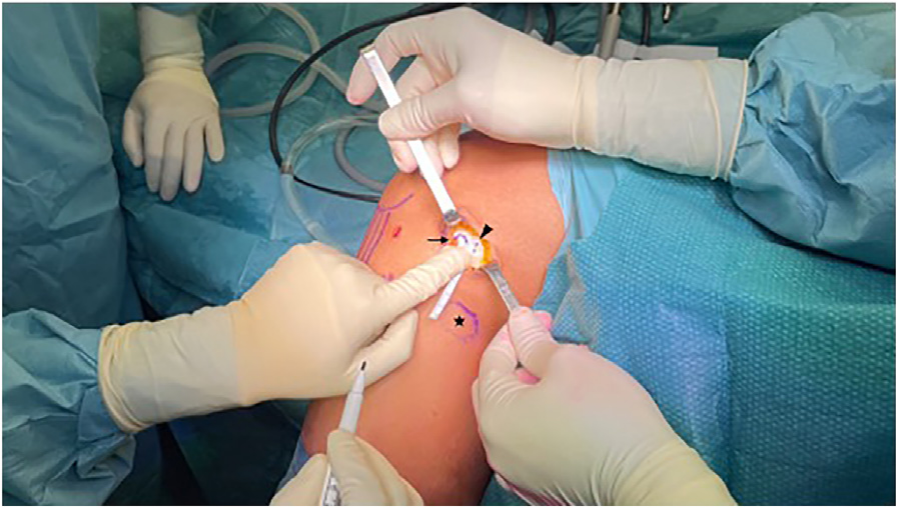
ALL femoral insertion. Lateral aspect of the femur: the fibular head (star) and the lateral epicondyle are identified (arrow). The femoral insertion of the ALL is then marked, just proximal and posterior to the lateral epicondyle (arrowhead). ALL, antero-lateral ligament.

A 3-cm stab incision is performed, centred on the lateral epicondyle, along the course of the fascia lata fibers. After making a small incision along the fibers of the fascia lata, the insertion of the ALL is identified just proximal and posterior to the lateral epicondyle, as previously described^6^ (Fig 2).

The outside-in guide for the femoral tunnel is placed so that the intra-articular part is positioned at the Isometric, Direct Fibers, Eccentric, Anatomic, Low Tension.^15^ insertion of the ACL, and the extraarticular access is located at the femoral insertion of the ALL. A guide pin is placed under arthroscopic visualization, and the tunnel is drilled at the proper diameter matching the graft dimension.

The tibial tunnel is then drilled using the proper guide aiming for a tunnel angled ∼20° medially in the axial plane and 55° inferior in the sagittal plane. Guide pin is inserted, anatomical position is arthroscopically checked, as well as potential extension impingement; then, the tunnel is drilled at the proper diameter matching the graft dimension.

### Graft Passage and Fixation of the ACL Graft

**Fig 3:**
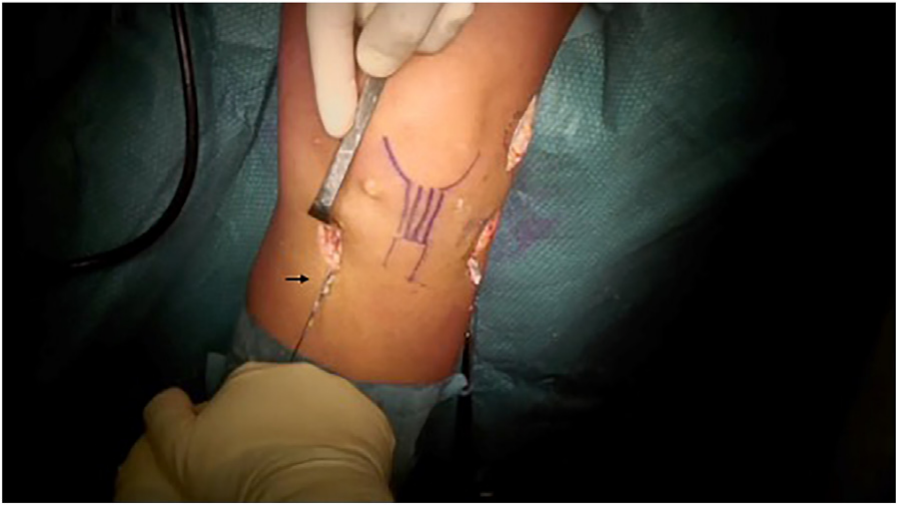
Tensioning of the ACL on the tibial side. In case of necessity, the distal suture (arrow) can be used for retensioning of the ACL from the tibial side to add tibial fixation. This can provide additional tension and backup fixation in case of a slack ACL graft. ACL, anterior cruciate ligament.

The graft is grasped from the free end and pushed into the articulation from the tibial tunnel. An endoscopic grasper is then passed from outside into the femoral tunnel to grasp the proximal loop of the graft that is then pulled outside the knee. With the knee at 30° of flexion and neutral rotation, the graft is slowly pulled from the femoral side and fixed with an interference screw matching the dimension of the tunnel. At the end of the procedure, proper tensioning of the ACL portion of the graft is arthroscopically probed both at 90° and in midflexion. If additional tension is required, the tibial portion of the ACL graft can be pulled by the distal whipstitch, and tibial fixation can be added (Fig 3).

### Fixation of the ALL Graft

**Fig 4:**
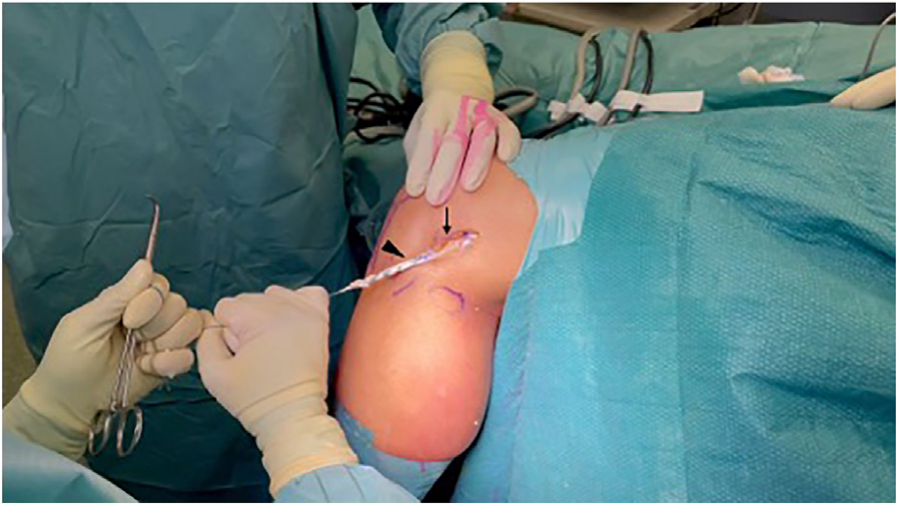
Passage of the graft under the LCL. From the same approach window of the lateral epicondyle, the LCL is identified (arrow), and a minimal dissection is performed at its anterior and posterior edges. The graft is then shuttled under the LCL from posterior to anterior (arrowhead). LCL, lateral collateral ligament.

A vertical small incision is performed on the anterolateral aspect of the tibia, halfway between Gerdy’s tubercle and the fibular head, centred at a point ∼10 mm below the articular surface. The LCL is identified and, with the help of a curved dissecting forcep, the graft is passed underneath it. This is a unique feature of the present technique, aiming to obtain a structure that minimizes lateral compartment overconstrain, as reported by recent biomechanical comparison tests^9^ (Fig 4).

The graft is then passed from the femoral tunnel to the tibial insertion, beneath the fascia latae.

Tibial anatomical insertion can be checked with fluoroscopy, if required. After exposing the tibial bony surface, the graft is fixed with a low-profile staple at proper tensioning with the knee in full extension and neutral rotation (Video 1).

### Postoperative Rehabilitation Protocol

The knee is kept in a brace for the first 4 weeks, allowing 0 to 90° flexion for the first 2 weeks and then complete motion. A partial progressive weight-bearing protocol was adopted, aiming to reach full weight bearing in 2 weeks. Early rehabilitation is begun focusing on quadriceps isometric strength and activation and gaining full extension; then it progresses in a specialized centre with a standard protocol for ACL rehabilitation. Gradual return to sport is allowed starting from 5 months for nonpivoting sport and at 6 to 9 months for pivoting and contact sports. Table 1 shows the pearls and pitfalls of this procedure, and Table 2 shows its advantages and disadvantages.

**Table 1:** Pearls and Pitfalls

Pearls
• Stitch the two tendons together both at the free end near the distal insertion, for backup tensioning of the graft.
• Insert the femoral socket screw wire before passing the graft to avoid tendon entanglement with the screw.
• Use curved forceps to pass the graft beneath the lateral collateral ligament (LCL). Minimal dissection at the anterior and posterior edge of LCL helps in passing the graft.
• At the end of the procedure, if the tension of the ACL graft is not satisfactory, additional tension and tibial fixation may be added.

PITFALLS
• Leaving intact the distal side insertions of the gracilis and semitendinosus may alter the tension distribution of the ACL graft at its distal point of fixation.
• Fixing the ALL graft with the tibia in external rotation may result in lateral compartment overconstraint.

**Table 2:** Advantages and Disadvantages

Advantages
• Vascularization of the graft is favored by maintaining the distal insertion of the hamstrings intact.
• Surgeon is able to simultaneously perform an anatomical reconstruction of both ACL and ALL, using only 1 graft.
• Evolving the “over the top” technique concept, this procedure adds the anatomical position and the intraosseus fixation of the graft.
• Only one tibial and one femoral tunnel is required, thus avoiding any concerns for tunnel collision.
• The procedure is straightforward and fast, with short surgical time.

Disadvantages
• The tension of the neo-ACL must be assessed to avoid a slack graft.
• The passage beneath the LCL of a big graft is sometimes tricky, needing minimal dissection of its edges.
• During the first steps of the learning curve, the lateral surgical approach should be extended, to avoid femoral tunnel malpositioning.

## Discussion

This study aimed to present our personal technique for combined reconstruction of ACL and ALL with single-stranded autologous GR and ST using a single tibial and femoral tunnel.

The presence of an ALL ligament was first hypothesized in 1879 by Segond, who identified the “so-called” avulsion fracture, but for many years, this anatomical structure remained largely underestimated. After consistent anatomical demonstration of the presence of this ligament^5,4^ and studies that confirmed the biomechanical importance of the entire ALC,^14^^,^^16^ the surgical interest toward ALL reconstruction and LET is growing.^10^^,^^17^ Moreover, there is now growing evidence confirming that addressing ALC complex by combined ALL reconstruction or performing LET reduces ACL ruptures^13^^,^^18^ and protects meniscal repairing procedures.^19^ Chahla et al.^20^ in 2016 described an anatomical isolate ALL reconstruction technique using a semitendinosus tendon allograft. This technique requires both a tibial and a femoral tunnel each made in a closed-socket fashion to prevent collision with ACL tunnels. In fact, the risk of tunnel collision represents a major issue, also given the fact that ACL revision surgery is one of the most diffuse indications for ALL reconstruction.^21^

Different authors described similar techniques using a 3-folded ST and single-stranded GR for combined reconstruction of ACL and ALL.^22^^,^^23^ These both require two confluent tunnels on the anterolateral aspect of the tibia that are time consuming and tricky.

With our technique, the correct femoral anatomical position of the ALL can be consistently reproduced by pointing the external entry point of the out-in femoral guide just proximal and posterior to the lateral epicondyle. In addition, the use of the same femoral socket adopted for the ACL reconstruction avoids any risk of femoral tunnel collision and saves time, making the whole procedure faster.

This position has demonstrated to reproduce the correct nonisometry of the ALL that is tight in extension and internal rotation at 20° and loose in flexion at 90° and 120°, as reported by different authors, and accepted by ALL Expert Group Consensus.^6^^,^^12^^,^^16^

In addition, a unique characteristic of our technique is the passage of the graft beneath the LCL, which prevents flexion overconstraint, as previously described.^9^

One of the potential criticisms to this technique is that a double-strand graft could be too thin to address the function of an ACL. We consistently found graft ranging from 7.0 mm to 7.5 mm of diameter. Although this seems apparently small to be an ACL graft, the extra-articular portion of the graft provides substantial additional anteroposterior and rotatory stability with his increased lever arm. Moreover, similar graft dimension has long been adopted for the “over-the-top” technique, with good results.^24^

In conclusion, this technique acquires some of the positive features of the “over the top” reconstruction, which has demonstrated good results at very long-term follow-up.^25^ In addition, this procedure combines a complete anatomical reconstruction of both the ACL and the ALL, with the advantages of the intraosseous fixation of the graft that provides superior mechanical properties and graft integration.^26^

## Supplementary Data

ICMJE author disclosure forms

Video 1 This video demonstrates our surgical technique for combined anatomic anterior cruciate ligament (ACL) and antero-lateral ligament (ALL) reconstruction. The knee is positioned, and examination under anaesthesia is performed confirming high-grade pivot shift. Medial and lateral landmarks are signed. A small anteromedial incision is performed, and semitendinosus and gracilis tendons are harvested using an open stripper. Care is taken to leave the distal insertion of both tendons intact. The two tendons are then whipstitched together at both extremities with high-resistance wires. Diameter is measured, and preparation of the graft is completed. On the lateral aspect of the knee, lateral femoral epicondyle, Gerdy’s tubercle and fibular head are identified and marked. A 3-cm incision is performed just posterior to the lateral epicondyle. Fascia lata is incised, and the anatomical femoral insertion of the anterolateral ligament is identified, proximal and posterior to the lateral epicondyle. With the help of an assistant, using an outside-in technique, a guide pin is positioned from the femoral insertion of the ALL to the femoral footprint of the ACL, and a tunnel of appropriate diameter is drilled. Tibial tunnel is then drilled using a standard technique. After positioning of the screw wire in the femoral socket, the graft is shuttled with two graspers through the tibial and femoral tunnels. After cyclic loading, femoral fixation is performed with the knee at 30° of flexion and neutral rotation. Lateral collateral ligament is identified and isolated, with the help of a curved forcep. The graft is then shuttled beneath the collateral ligament, from posterior to anterior. This can be done directly or with the help of a shuttling wire. A small vertical incision is performed, halfway between the Gerdy’s tubercle and the fibular head. With the help of a needle, the anatomic tibial insertion of the ALL is identified, about 10 mm below the articular surface. Fluoroscopy can be used for confirmation. The graft is passed underneath the fascia latae. Fixation of the graft in the tibial anatomical ALL insertion is performed with a low-profile staple, keeping the knee in full extension and neutral rotation. Finally, proper tension of the graft is demonstrated. If additional tension is required, the tibial portion of the ACL can be pulled using the distal whipstitch, and the tibial fixation can be added using an interference screw.

## References

[1] Ardern C.L., Webster K.E., Taylor N.F., Feller J.A. (2011). Return to sport following anterior cruciate ligament reconstruction surgery: a systematic review and meta-analysis of the state of play. Br J Sports Med.

[2] Crawford S.N., Waterman B.R., Lubowitz J.H. (2013). Long-term failure of anterior cruciate ligament reconstruction. Arthrosc J Arthrosc Relat Surg.

[3] Samitier G., Marcano A.I., Alentorn-Geli E., Cugat R., Farmer K.W., Moser M.W. (2015). Failure of anterior cruciate ligament reconstruction. Arch Bone Jt Surg.

[4] Daggett M., Busch K., Sonnery-Cottet B. (2016). Surgical dissection of the anterolateral ligament. Arthrosc Tech.

[5] Claes S., Vereecke E., Maes M., Victor J., Verdonk P., Belleman J. (2013). Anatomy of the anterolateral ligament of the knee. J Anat.

[6] Daggett M., Ockuly A.C., Cullen M. (2016). Femoral origin of the anterolateral ligament: An anatomic analysis. Arthroscopy.

[7] Dodds A.L., Halewood C., Gupte C.M., Williams A., Amis A.A. (2014). The anterolateral ligament: Anatomy, length changes and association with the Segond fracture. Bone Joint J.

[8] Chahla J., Moatshe G., Geeslin A.G., LaPrade R.F. (2017). Biomechanical role of lateral structures in controlling anterolateral rotatory laxity: The anterolateral ligament. Oper Tech Orthop.

[9] Inderhaug E., Stephen J.M., Williams A., Amis A.A. (2017). Biomechanical comparison of anterolateral procedures combined with anterior cruciate ligament reconstruction. Am J Sports Med.

[10] Sonnery-Cottet B., Thaunat M., Freychet B., Pupim B.H.B., Murphy C.G., Claes 3 Steven (2015). Outcome of a combined anterior cruciate ligament and anterolateral ligament reconstruction technique with a minimum 2-year follow-up. Am J Sports Med.

[11] Ueki H., Nakagawa Y., Ohara T. (2018). Risk factors for residual pivot shift after anterior cruciate ligament reconstruction: Data from the MAKS group. Knee Surg Sport Traumatol Arthrosc.

[12] Sonnery-Cottet B., Daggett M., Fayard J.-M., Ferretti A. (2017). Anterolateral ligament expert group consensus paper on the management of internal rotation and instability of the anterior cruciate ligament—deficient knee. J Orthop Traumatol.

[13] Sonnery-Cottet B., Saithna A., Cavalier M. (2017). Anterolateral ligament reconstruction is associated with significantly reduced ACL graft rupture rates at a minimum follow-up of 2 years: A prospective comparative study of 502 patients from the SANTI Study Group. Am J Sports Med.

[14] Kennedy M.I., Claes S., Freitas Fuso F.A. (2015). The anterolateral ligament: An anatomic, radiographic, and biomechanical analysis. Am J Sports Med.

[15] Pearle A.D., McAllister D.H.S. (2015). Rationale for strategic graft placement in anterior cruciate ligament reconstruction: I.D.E.A.L. femoral tunnel position. Am J Orthop (Belle Mead NJ).

[16] Parsons E.M., Gee A.O., Spiekerman C., Cavanagh P.R. (2015). The biomechanical function of the anterolateral ligament of the knee. Am J Sports Med.

[17] Delaloye J.R., Hartog C., Blatter S. (2020). Anterolateral ligament reconstruction and modified Lemaire lateral extra-articular tenodesis similarly improve knee stability after anterior cruciate ligament reconstruction: A Biomechanical Study. Arthroscopy.

[18] Getgood A.M.J., Bryant D.M., Litchfield R. (2020). Lateral extra-articular tenodesis reduces failure of hamstring tendon autograft anterior cruciate ligament reconstruction: 2-year outcomes from the STABILITY study randomized clinical trial. Am J Sports Med.

[19] Sonnery-Cottet B., Saithna A., Blakeney W.G. (2018). Anterolateral ligament reconstruction protects the repaired medial meniscus: A comparative study of 383 anterior cruciate ligament reconstructions from the SANTI Study Group with a minimum follow-up of 2 years. Am J Sports Med.

[20] Chahla J., Menge T.J., Mitchell J.J., Dean C.S., LaPrade R.F. (2016). Anterolateral ligament reconstruction technique: An anatomic-based approach. Arthrosc Tech.

[21] Jaecker V., Herbort M., Bouillon B., Günther D., Shafizadeh S. (2020). High incidence of tunnel collision in combined ACL reconstruction and LET. Orthop J Sport Med.

[22] Jankovic S., Vrgoc G., Vuletic F., Ivkovic A. (2021). Modified technique for combined reconstruction of anterior cruciate ligament and anterolateral ligament. Arthrosc Tech.

[23] Sonnery-Cottet B., Daggett M., Helito C.P., Fayard J.-M., Thaunat M. (2016). Combined anterior cruciate ligament and anterolateral ligament reconstruction. Arthrosc Tech.

[24] Marcacci M, Zaffagnini S, Iacono F, Surgery MN-K, Sports undefined, 1998 undefined. In: Arthroscopic intra-and extra-articular anterior cruciate ligament reconstruction with gracilis and semitendinosus tendons. New York: Springer. October 19, 2021. 10.1007/s001670050075 9604189

[25] Zaffagnini S., Marcheggiani Muccioli G.M., Grassi A. (2017). Over-the-top ACL reconstruction Plus Extra-articular Lateral Tenodesis With Hamstring Tendon Grafts: Prospective evaluation with 20-year minimum follow-up. Am J Sports Med.

[26] Deehan D.J., Cawston T.E. (2005). The biology of intergration of the anterior cruciate ligament. J Bone Jt Surg Ser B.

